# A Unified Ranking Model for Evaluating Snatch and Clean and Jerk Performances Across Body Mass and Sex

**DOI:** 10.3390/sports14070294

**Published:** 2026-07-10

**Authors:** Marianne Huebner, Rayleigh Lei

**Affiliations:** 1 Department of Statistics and Probability, Michigan State University, East Lansing, MI 48824, USA; 2 Department of Kinesiology, Michigan State University, East Lansing, MI 48824, USA; 3 Center for Statistical Training and Consulting, Michigan State University, East Lansing, MI 48824, USA; leirayle@msu.edu

**Keywords:** body mass effect, weightlifting, performance ranking, competitions, sport, sex differences

## Abstract

In Olympic-style weightlifting males outperform females and heavier weightlifters lift more weight than athletes with lighter body mass. Traditional ranking models in weightlifting are based on total weight lifted and do not permit direct comparison of individual lift performances across body mass and sex. The aim of this study was to model performance in the snatch and clean and jerk and to develop a unified ranking system for comparisons across body mass and sex. Data from 3412 performances at IWF Senior World Championships and Olympics (2019–2025) were analyzed. The median sex gap was 30.7% for the snatch and 29.3% for the clean and jerk, with smaller differences at lower body mass. Peak performance occurred at 25.4 years for both lifts. Generalized additive models accounting for location, scale, and shape of the performance distribution were used to estimate standardized scores. Scaling individual lifts onto a common metric allows for meaningful evaluation of athletes even in the presence of unsuccessful attempts in one type of lift and can illustrate performance profiles. This approach supports performance assessment, athlete monitoring, and mixed-team rankings which is particularly relevant in emerging competition formats that emphasize lift-specific contributions and mixed-sex participation.

## 1. Introduction

Olympic-style weightlifting performance consists of two technically and physiologically distinct lifts: the snatch and the clean and jerk. These lifts are explosive, multi-joint movements requiring strength, power, and coordination [1,2,3]. The snatch is a one-part movement with a wide grip on the bar, lifting the bar from the floor to overhead, with barbell velocities reaching up to 3.0 m·s^−1^. The clean and jerk is a two-part movement with a narrow grip on the bar, lifting the bar first to the shoulder, and becoming motionless before the bar is vertically driven off the shoulder and held overhead with fully extended arms involving a higher load than in the snatch. The sum of the best snatch (of three) and best clean and jerk (of three) is referred to as the ‘total’ and is the basis for podium awards and ranking of athletes. Performances in the individual lifts are also recognized, with medals awarded separately for the snatch and clean and jerk within body weight categories in some competitions, although they do not currently determine placement in the Olympic Games.

A challenge in weightlifting is the comparison of performances across athletes differing in body mass and sex. Typically, heavier athletes lift more weight, and males outperform females. Established approaches such as Sinclair points or Q-points adjust performances across body mass, but these methods were developed for the total weight lifted and thus cannot be directly applied to the individual lifts [4,5]. The snatch requires greater barbell velocity, technical precision, and coordination, whereas the clean and jerk involves higher loads and greater force production and strength [2,3]. Therefore, aggregating both lifts into a single total for body mass-adjusted rankings may obscure meaningful differences in athlete capabilities, limit performance comparisons, and exclude athletes with unsuccessful attempts in one type of lift.

Emerging competition structures, including mixed-sex team formats require performance metrics that allow direct comparison across sexes and potentially across individual lifts [6,7,8]. Previous work developed a scaling method to place male and female performances on a common scale for the total [9], but this does not apply to individual lifts. Some attempts have been made to choose coefficients to scale female performances to match those of males in competitions that are applied to individual lifts [6,10].

In addition to scaling across body mass and sex, understanding the age at which athletes reach peak performance is essential for athlete development and competition planning. Previous research has identified peak performance in weightlifting to occur in the mid-twenties for the total, with variation across body mass and developmental stages [11]. However, it is unclear whether age at peak performance differs between the snatch and clean and jerk, given their distinct physiological demands.

The purpose of this study was to estimate the effect of body mass on the weight lifted in the snatch and in the clean and jerk to rank weightlifting performances in these two lifts separately across body mass and on the same scale for males and females. Secondary aims were the estimation of the age at peak performance for each lift and to estimate the sex gap across body mass. By establishing scaled performance metrics for individual lifts, this work provides a foundation for more nuanced performance evaluation and supports emerging competition formats that require integration of performances across athletes, sexes, or disciplines.

## 2. Materials and Methods

### 2.1. Data

Data from the IWF Senior World Championships and Olympics data for 2019–2025 were used to develop the model which includes information on competition date, athlete’s name, sex, age, body mass, best snatch and best clean and jerk. The age category “senior” in weightlifting comprises athletes aged 15 and older. This dataset included 3412 competition results from 2942 athletes, aged 15 to 41, from 141 countries. The time range was chosen because the IWF started partnering with the International Testing Agency (ITA) in 2019 to manage its anti-doping program [12]. All athletes with anti-doping rule violations identified through IWF and ITA sanction records were excluded. Our findings are reported according to the STROBE statement [13]. For the model development athletes with at least one successful lift were included in the models for the corresponding lift. Thus, athletes who did not post a total could be included in one of these models.

### 2.2. Statistical Methods

#### 2.2.1. Scaled Models for Snatch and Clean and Jerk

Generalized additive models for location, scale, and shape (GAMLSS) [14] were used to estimate the distribution of the lifted weight for given body mass, separately for each sex and for snatch and clean and jerk. GAMLSS are regression models that account for nonlinearity, heteroscedasticity, and skewness, where heteroscedasticity indicates nonconstant error variance and skewness is an asymmetric error distribution. For this analysis, a Box–Cox Cole–Green (BCCG) distribution was fitted using generalized additive models for location, scale, and shape (GAMLSS). The location (μ), scale (σ), and shape (ν) parameters of the BCCG distribution correspond to the median, coefficient of variation, and Box–Cox power transformation used to account for skewness in the distribution, respectively [14,15]. Median performance (μ) was modeled as a smooth function of body mass using penalized B-splines (pb), and the coefficient of variation (σ) was similarly modeled as a smooth function of body mass [16]. The skewness parameter (ν) was modeled as a linear function of log-transformed body mass to accommodate changes in distributional asymmetry across the body mass range. Body mass-specific centile curves were subsequently derived from the fitted BCCG model.

(1) $$\begin{array}{c} log(\mu )={\;a}_{\mu }+\mathrm{pb}(\mathrm{body}\;\mathrm{mass})\\ \log(\sigma )=a_{\sigma }+\mathrm{pb}(\mathrm{body}\;\mathrm{mass})\\ \;\upsilon =a_{\upsilon }+\log\;\mathrm{body}\;\mathrm{mass}\; \end{array}$$

To calculate GAMX points (=Generalized Additive Model by seX) everyone’s weight lifted (best snatch and best clean and jerk) was replaced by its standardized residual or ‘z-score’ as obtained from the model, which adjusts for differences in body mass. This has the advantage that it puts males and females on the same scale. The z-score was then converted to GAMX points using the following formula:

(2) GAMX=100∗z-score+1000

where the offset of 1000 ensures that an individual’s GAMX points cannot be confused with their weight lifted in kilograms. In case of ties in competitions the winner of one of the lifts or the total is determined by who has lifted the weight first on the platform. For the overall ranking it is desirable to have a point system that separates performances. A difference in body mass will matter and a sufficiently large scale may avoid ties even with rounding.

#### 2.2.2. Model Evaluation

Candidate GAMLSS models based on the Box–Cox Cole–Green (BCCG), Box–Cox Cole–Green original (BCCGo), Box–Cox power exponential (BCPE), and Box–Cox t (BCT) distributions were evaluated. Models using body mass and log-transformed body mass as predictor functions were compared. Model selection was guided by the generalized Akaike information criterion (GAIC), worm plots, Q-statistics of normalized quantile residuals, and visual inspection of the resulting centile curves. Worm plots were examined for systematic linear, quadratic, cubic, and quartic departures, corresponding to potential misspecification of the location (μ), scale (σ), skewness (ν), and kurtosis (τ) components of the fitted distribution, respectively. Q-statistics were used to formally assess these distributional components across groups of observations. Calibration of the fitted centiles was evaluated by comparing the observed proportion of athletes falling below selected centiles with the expected proportions. Final model selection was based on overall diagnostic performance, model parsimony, and the smoothness and biological plausibility of the derived centile curves.

#### 2.2.3. Sex Gap

The sex gap was calculated as 100 × (male − female performance)/male performance where the performance is predicted from the model (1) for the range of body mass. Positive values indicate lower female performances relative to male performances, expressed as a percentage [17].

#### 2.2.4. Model for Age at Peak Performance

To estimate the age at peak performance, quantile regression models [18] were used modeling the 90th percentile of the performance as a quadratic function of age. Confidence intervals were estimated with bootstrap sampling. Quantile regression models are an extension of traditional regression models estimating curves for the median, quartiles, or other quantiles. The model for the peak performance was

(3) $$\mathrm{GAMX}\;=a*\mathrm{Age}+b*{\mathrm{Age}}^{2}\;\;+c\;$$

using the exact age at the first day of the competition, where GAMX refers to the performance in the snatch (GAMX-S) and clean and jerk (GAMX-J) after scaling across body mass and sex. There were 1000 iterations for random samples of 500 athletes drawn at each iteration to estimate 95% confidence intervals for the 90th percentile. Quantile regression models have the advantage that results are less likely to be skewed due to outliers, and it is possible to estimate peak age at different percentiles.

All analyses were performed using the statistical software package R version 4.5.1 [19]. GAMLSS was fitted using the gamlss package version 5.4-12 [14]. The quantile regression package version 6.1 was used to estimate peak age.

## 3. Results

There were 3412 IWF Senior World Championships and Olympics results (48% females), with ages of 15–41 years (Table 1). Overall, among the snatches 7.9% were not valid, and among the clean and jerks 11.9% were not valid, i.e., the athletes could not maintain control of the barbell, or had a rule violation per IWF Technical and Competition rules [20].

The largest age group in these senior world championships is 21 to 30 years old (74%). The body mass ranges from 43 to 155 kg for females and from 53 to 188 kg for males with a multimodal distribution due to athletes striving to be at the upper end of a body mass category.

### 3.1. Performance Across Body Mass in Snatch and Clean and Jerk and Sex Gap

**Figure 1:**
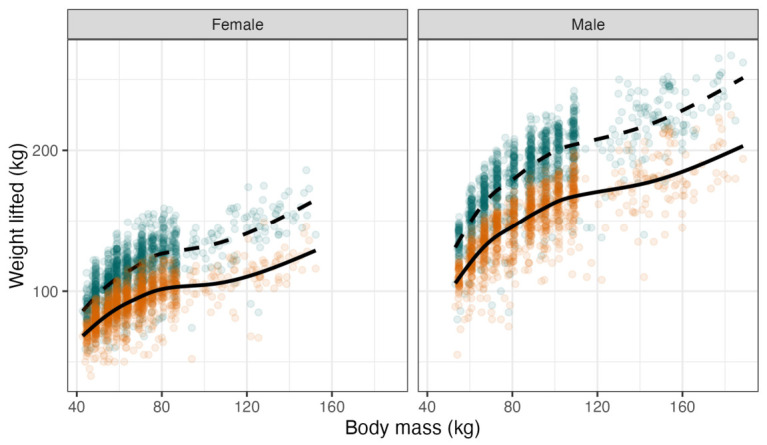
Individual snatch (orange) and clean and jerk (green) performances across body mass for both sexes. Cubic regression lines are fitted to the data points: solid lines for female and dashed lines for males.

Athletes with higher body mass lift more weight compared to athletes with lower body mass (Figure 1). The median sex gap is 30.7% and 29.3% in the snatch and clean and jerk, respectively (Table 2). The sex gap is larger for higher body mass than for lower body mass. For example, the sex gap is 22% at 50 kg, 26% at 60 kg, 30% at 70 kg, and 31% at 80 kg and is similar for both lifts.

### 3.2. Scaled Points for Snatch and Clean and Jerk

**Figure 2:**
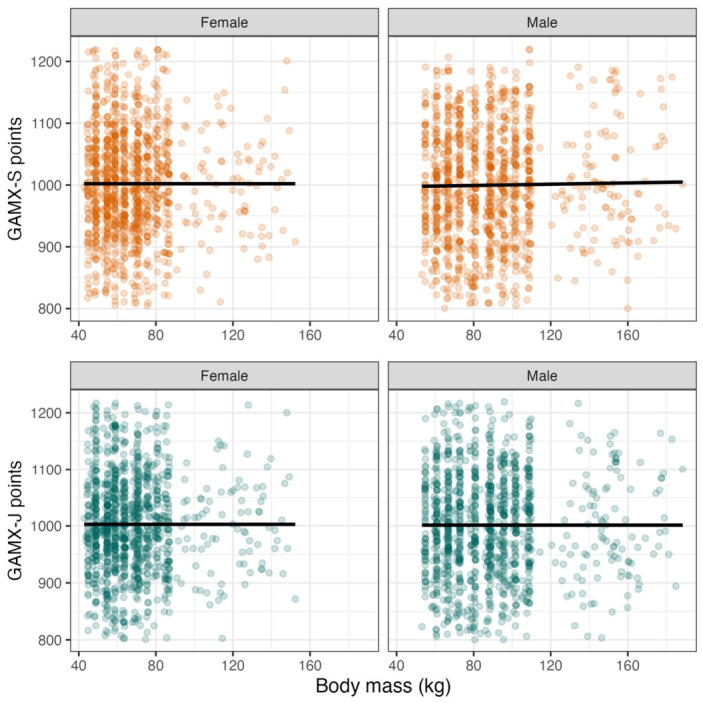
Points for snatch (GAMX-S, orange) and clean & jerk (GAMX-J, green) across body mass for females and males.

GAMX-S and GAMX-J points were calculated from Model 1 across body mass and sex and scaled with Model 2. Scatterplots of individual GAMX points are shown in Figure 2. This shows that the resulting points are on the same scale for males and females. The fitted cubic regression splines across body mass are horizontal with a median of 1000 showing that there are no body mass-related trends.

Among the candidate distributions, the BCCG model provided the most appropriate balance of fit, stability, and interpretability. Although alternative distributions occasionally yielded lower GAIC values and modest improvements in some diagnostic statistics, they produced centile curves that were similar to those obtained with the BCCG model and did not materially alter the substantive findings. Consequently, the BCCG distribution was retained to provide a consistent modeling framework across sexes and lifts.

Diagnostic evaluations indicated adequate model fits. For the male models, Q-statistics identified residual departures primarily in the location component, with no evidence of substantial misspecification of the scale, skewness, or kurtosis components. For the female models, Q-statistics indicated residual departures in the location component together with evidence of residual skewness and borderline kurtosis misspecification. However, the magnitude of these departures was modest, and worm plots showed only minor systematic deviations from the expected pattern. Overall, the fitted centile curves remained smooth, stable, and biologically plausible, supporting their use as reference standards.

**Figure 3:**
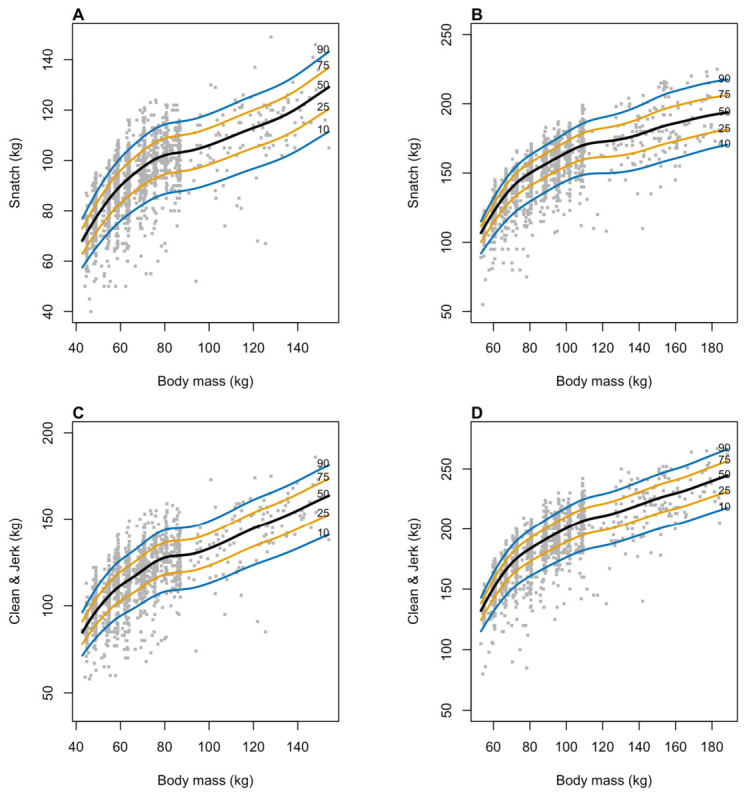
Body mass-specific reference centile curves for weightlifting performance. Shown are the 10th, 25th, 50th, 75th, and 90th centiles estimated from GAMLSS models for (**A**) women’s snatch, (**B**) men’s snatch, (**C**) women’s clean and jerk, and (**D**) men’s clean and jerk.

Body mass-specific centile curves demonstrated a positive, nonlinear relationship between body mass and performance for both the snatch and clean and jerk (Figure 3). Performance increased rapidly at lower body masses before increasing more gradually among heavier athletes. The centile curves were more closely spaced at lower body masses and became progressively wider with increasing body mass, indicating lower variability in performance among lighter athletes and greater heterogeneity in performance among heavier athletes. Overall, the fitted centile curves were smooth and monotonic across the body mass range. In women, the clean and jerk centile curves exhibited a modest flattening at the onset of the heaviest body mass categories before increasing again at higher body masses (Figure 3C). This pattern was evident across multiple centiles, suggesting that the association between body mass and performance was less regular in the heaviest women’s categories than in the corresponding men’s categories.

### 3.3. Performance Profiles of Individual Athletes

**Figure 4:**
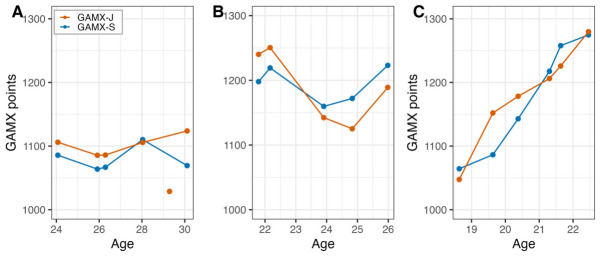
Comparison of snatch and clean & jerk performances across age with GAMX-S and GAMX-J points for three athletes ((**A**) female, (**B**) male, (**C**) female).

The relative performance in snatch and clean and jerk can be compared for specific athletes over time. For example, GAMX points are shown at different ages for three athletes competing in senior world championships in Figure 4. Athlete A consistently performed above the average with higher points for the clean and jerk than for the snatch. At age 29, athlete A missed all three snatches but was able to post the first attempt in the clean and jerk. Her body weight category was 77 kg except at ages 26 and 28 when it was 86 kg. Athlete B initially performed better in the clean and jerk and then performed better in the snatch. At age 23 this athlete did not compete/withdrew from competition. His body weight category was 110 kg except for age 23 when it was +110 kg. Athlete C improved over time in both lifts. Her body weight category was 77 kg at all ages. Body mass was categorized according to the 2025 IWF body weight categories.

### 3.4. Age at Peak Performance

**Figure 5:**
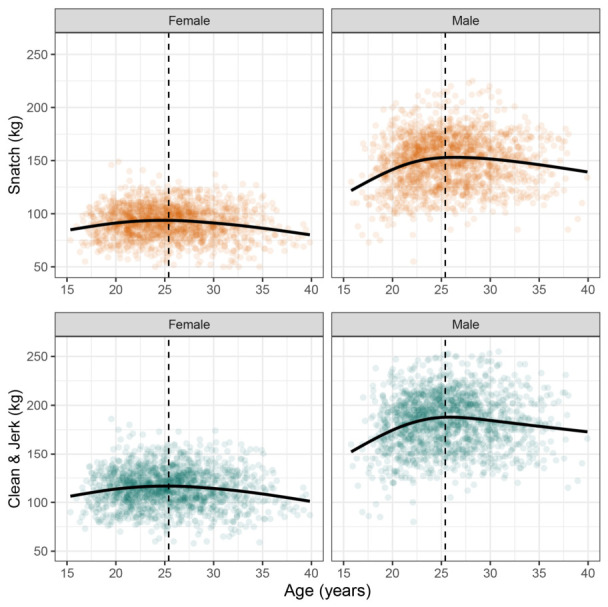
Age-related trends in snatch (orange) and clean and jerk (green) performance for females and males with a vertical reference line at 25.4 years.

The scaled performances for snatch and clean and jerk were adjusted for body mass, but not for age. Elite athletes in the 90th percentile at world championships reached their peak performance in the snatch, as measured by GAMX-S, at 25.4 years (95% CI: 20.5, 27.6). The age at peak performance for the clean and jerk, as measured by GAMX-J, was 25.4 years (95% CI: 20.6, 27.5). For heavier body mass (females > 80 kg, males > 110 kg), the peak ages were slightly higher, 26.0 years (95% CI: 24.2, 27.0) for the snatch and 26.7 years (95% CI: 24.6, 27.8) for the clean and jerk. There were no sex differences in peak age in the snatch (95% CI were 23.1 to 26.1 for males and 25.1 to 26.3 for females) or the clean and jerk (95% CI were 23.2 to 28.0 for males and 25.2 to 26.8 for females). Scatterplots with individuals’ best snatch and best clean and jerks [kg] as a function of exact age, stratified by sex are shown in Figure 5. Solid lines indicate fitted cubic regression splines to capture nonlinear age-related trends.

## 4. Discussion

Using results from the IWF Senior World Championships and the Olympics (2019–2025), we developed a unified approach for evaluating and ranking performances in the snatch and clean and jerk separately, enabling meaningful comparison of athletes across lifts, body mass, and sex. By placing performances from both lifts on a common scale, the model allows athletes to be evaluated even when only one valid lift is achieved. It provides a consistent basis for integrating individual lift performance into combined or team-based scoring systems.

In contrast, traditional rankings across body mass based on the combined total are limited in their ability to capture individual performance profiles [4,5]. In elite competition, a proportion of attempts in both lifts are recorded as “no lifts,” indicating unsuccessful execution due to technical errors, loss of control, or rule violations [20]. Under current rules, athletes who fail to obtain a valid result in either lift receive a total of zero. Thus, traditional rankings may obscure meaningful differences between the snatch and clean and jerk and exclude athletes with unsuccessful attempts in one lift, limiting their applicability in contexts where individual lift contributions are relevant.

Recent developments in the Olympic Movement provide a strong rationale for the development of unified performance metrics that enable comparisons across sex and individual lifts. Mixed-team events have been introduced across a wide range of Olympic sports, including judo, athletics, swimming, triathlon relays, archery mixed team events, and shooting disciplines [21]. The International Olympic Committee is actively promoting mixed-gender events as a means of fostering inclusion, cooperation, and innovation in competition formats [22]. These developments are aligned with the IWF’s stated goal of strengthening its position within the Olympic program by adapting to contemporary expectations regarding inclusivity and fairness [7,23]. The GAMX scoring systems provide a methodological framework that is compatible with these evolving competition structures and are consistent with the previously developed scoring system for the total as is needed for classic competitions [9]. By placing male and female performances on a common scale and allowing separate evaluation of the snatch and clean and jerk, the model enables objective comparisons that are essential for mixed-team formats and specialized competition roles.

### 4.1. Sex Differences

Sex differences in sport performance in weightlifting and other sports can be attributed to hormones and epigenetics and other biological determinants [24,25,26]. In our study we estimated the median sex gap across body mass to be 30.7% and 29.3% in the snatch and clean and jerk, respectively. The sex gap ranged from 20% for lower body mass to 30% for larger body mass, for both lifts. Prior work only considered the sex gap in world records in the total and limited to a body mass category that was the same for males and females [4,27]. However, these studies did not consider differences in body mass or that world records may not be representative even for elite athletes. The sex gap in weightlifting was higher than in aerobic events, such as running or swimming, where it can be less than 10% [28]. In iron-triathlon athletes, the sex gaps were 24.8% in cycling and 8.5% in swimming [29]. An important contribution of the GAMX system is its ability to evaluate female weightlifting performances using a framework that places women and men on a common scale while explicitly accounting for sex-specific performance distributions. Rather than applying arbitrary adjustment factors, the model derives standardized scores from observed international performance data for each sex. This allows exceptional female performances to be recognized relative to the elite female athlete population while also enabling meaningful comparisons across sexes. Such a framework may enhance the visibility of female athletes in performance analyses, facilitate equitable athlete evaluation and selection procedures, and support emerging mixed-sex competition formats in which women contribute directly to team outcomes alongside men. Furthermore, because GAMX-S and GAMX-J evaluate the snatch and clean and jerk separately, the system provides additional opportunities to identify and recognize strengths in female athletes that may be obscured when only the total weight lifted is considered.

### 4.2. Age at Peak Performance

The age at peak performance in weightlifting was found to be 25.4 years. However, the relatively wide confidence intervals (20.5 to 27.5 years) suggest substantial uncertainty in the exact peak age, indicating that elite performance can be maintained across a range of ages within the mid-twenties. There were no differences in the peak age for snatch or clean and jerk, nor were there sex differences. This is consistent with earlier findings for the peak age in the total weight lifted derived using different statistical methods (quantile foliation) and for an earlier time period, 2013–2017 [30]. The previous study only considered the total weight lifted and limited the age range to less than 30 years. The peak age in weightlifting was similar to track and field where peak age was around 25 to 27 years, but could be higher in throwing disciplines [31,32]. In a systematic review, no sex differences in peak age were reported in different sports [31].

### 4.3. Strengths and Limitations

Body mass was the only indicator of the physique of the athlete; no other anthropometric variables were available. However, we were able to compare performance levels for athletes of different body mass which has not been established within track and field disciplines where throwers typically have large body mass, exceeding 100 kg [26,27].

Since competitions for senior athletes do not differentiate between ages, no attempt has been made to create age-adjusted GAMX-S and GAMX-J points in this paper. However, age-adjusted GAMX points have been developed for Masters for the total as well for snatch and clean and jerk [33]. Thus, it is feasible to expand this work to youth, juniors, and seniors, should competitions implement such rankings.

While higher performances of weightlifters may be possible at national or continental competitions, the choice of world championships ensures that performances are judged under uniform conditions that cannot be guaranteed in other events or circumstances. The model was developed from Senior World Championship and Olympic performances and therefore represents an international elite reference population. This approach is consistent with existing ranking systems in weightlifting, such as Sinclair and Q-points, which are derived from elite athletes but applied across all competition levels. The availability of model parameters, reference tables, an Excel calculator [33], and implementation in competition software facilitates broad application. Future research may examine the distribution of GAMX points for specific populations or competition levels.

Weightlifting body mass distributions are influenced by discrete weight categories, resulting in clustering of athletes near category limits. Although penalized B-splines within the GAMLSS framework help smooth local irregularities and reduce overfitting, spline estimates may still be influenced by the distribution of observations across body mass.

We excluded all athletes with anti-doping rule violations identified through official IWF and International Testing Agency sanction records. However, residual confounding due to undetected doping cannot be completely excluded because undetected doping cannot be ruled out.

Finally, the analyses were based on competition results collected over multiple years and were not designed as a longitudinal study of athlete development. Although some athletes contributed repeated observations, age-related findings should be interpreted as population-level patterns among elite competitors.

### 4.4. Practical Implications

The proposed GAMX scoring system provides a flexible framework for evaluating weightlifting performance across body mass, sex, and individual lifts, with several practical implications for athletes, coaches, and sport organizations.

GAMX-S and GAMX-J points allow athletes and coaches to track performance longitudinally in the snatch and clean and jerk separately, independent of body mass. This enables identification of relative strengths and weaknesses within athletes (for example, better performances in the clean and jerk than in the snatch), facilitating targeted training interventions and technique development. The use of standardized scores also allows benchmarking against an international reference population across body mass categories. By separating snatch and clean and jerk performance, GAMX points allow more precise evaluation of performance profiles and competition outcomes, including scenarios where athletes fail one lift but successfully complete the other.

Furthermore, by placing male and female performances on a unified scale, GAMX points provide an objective basis for evaluating athletes in mixed teams (example in Table 3). This facilitates fair comparisons in team selection, ranking, and scoring systems without requiring arbitrary scaling factors. Such an approach supports the increasing emphasis on gender equity and mixed-team competitions in international sport.

For example, a team consisting of four athletes, two women and two men, each performing a snatch or a clean and jerk, the team points can be calculated as follows.

The availability of a web application and Excel-based implementation facilitates immediate adoption by athletes, coaches, and national organizations. These tools allow real-time calculation of GAMX scores during training or competition, supporting decision-making in applied sport settings.

## 5. Conclusions

We developed a unified ranking model for evaluating snatch and clean and jerk performances that enables direct comparison of performances across body mass and sex. By scaling individual lifts onto a common metric, this approach captures distinct performance profiles and allows for meaningful evaluation of athletes even in the presence of unsuccessful attempts in one type of lift. In addition, we identified an age range of peak performance across both lifts although confidence intervals indicate considerable uncertainty regarding the precise age at which peak performance occurs. The proposed GAMX ranking system has practical applicability for athletes, coaches, and sport organizations, supporting performance monitoring, benchmarking, and selection decisions. It provides a methodological foundation for emerging competition formats, including mixed-sex team events and discipline-specific roles, where direct comparison across athletes and lifts is required. While age adjustment is not currently used in senior competitions, the framework can be extended to incorporate age effects, making it adaptable to Masters and youth categories. Overall, this work advances performance evaluation in weightlifting by enabling more precise, equitable, and flexible assessment of athletes, aligned with current practice and evolving competition structures.

## Figures and Tables

**Table 1:** Description of competition results by sex.

	Females N = 1642 Results (811 Athletes)	Males N = 1770 (827 Athletes)
Age, years (mean ± sd)	25.2 ± 4.5	25.4 ± 4.3
Body mass, kg (median, quartiles)	63.7 (55.0, 75.9)	87.5 (70.3, 101.5)
Age group		
15–17 years	2.0% (33)	1.0% (18)
18–20 years	12.3% (202)	10.3% (183)
21–30 years	72.5% (1190)	75.1% (1330)
31–41 years	13.2% (217)	13.5% (239)
Snatch, not valid	6.6% (109)	9.1% (161)
Clean & jerk, not valid	8.8% (144)	14.8% (262)

**Table 2:** Median and range of performances and sex gap.

	Females, kg	Males, kg	Sex Gap, %
Snatch	91 (40–149)	150 (55–225)	30.7 (18.4–35.9)
Clean & jerk	114 (58–186)	183 (80–267)	29.3 (20.6–33.8)

**Table 3:** Points for a team of four athletes, calculated as the sum of GAMX-S and GAMX-J points.

Athlete	Body Mass	Snatch	Clean & Jerk	GAMX-S or GAMX-J
A, female	58.9	95	---	1068
B, male	108.3	177	---	1055
C, female	132.3	---	160	1069
D, male	73.0	---	187	1093
**Team points**				**4285**

## Data Availability

The datasets for world championships can be accessed from the website of the International Weightlifting Federation (https://iwf.sport/results/results-by-events/). Athletes with sanctions are listed for international athletes (https://iwf.sport/anti-doping/sanctions/). A web application (https://huebner.shinyapps.io/GAMX_teams) and Excel workbook on the Open Science Framework [33] are available.

## Abbreviations

The following abbreviations are used in this manuscript:

IWF	International Weightlifting Federation
GAMX	Generalized additive models adjusted for sex
GAMX-S	GAMX for snatch
GAMX-J	GAMX for clean and jerk
